# Mast Cell Stabilization Alleviates Acute Lung Injury after Orthotopic Autologous Liver Transplantation in Rats by Downregulating Inflammation

**DOI:** 10.1371/journal.pone.0075262

**Published:** 2013-10-08

**Authors:** Ailan Zhang, Xinjin Chi, Gangjian Luo, Ziqing Hei, Hua Xia, Chenfang Luo, Yanling Wang, Xiaowen Mao, Zhengyuan Xia

**Affiliations:** 1 Department of Anesthesiology, The Third Affiliated Hospital of Sun Yat-sen University, Guangzhou City, People's Republic of China; 2 Department of Anesthesiology, the Affiliated Hospital of Luzhou Medical College, Luzhou City, People's Republic of China; 3 Department of Anesthesiology, Research Centre of Heart, Brain, Hormone and Healthy Aging, University of Hong Kong, Hong Kong SAR, People's Republic of China; University of Virginia, United States of America

## Abstract

**Background:**

Acute lung injury (ALI) is one of the most severe complications after orthotopic liver transplantation. Amplified inflammatory response after transplantation contributes to the process of ALI, but the mechanism underlying inflammation activation is not completely understood. We have demonstrated that mast cell stabilization attenuated inflammation and ALI in a rodent intestine ischemia/reperfusion model. We hypothesized that upregulation of inflammation triggered by mast cell activation may be involve in ALI after liver transplantation.

**Methods:**

Adult male Sprague–Dawley rats received orthotopic autologous liver transplantation (OALT) and were executed 4, 8, 16, and 24 h after OALT. The rats were pretreated with the mast cell stabilizers cromolyn sodium or ketotifen 15 min before OALT and executed 8 h after OALT. Lung tissues and arterial blood were collected to evaluate lung injury_._ β-hexosaminidase and mast cell tryptase levels were assessed to determine the activation of mast cells. Tumor necrosis factor α (TNF-α), interleukin (IL)-1β and IL-6 in serum and lung tissue were analyzed by enzyme-linked immunosorbent assay. Nuclear factor-kappa B (NF-κB) p65 translocation was assessed by Western blot.

**Results:**

The rats that underwent OALT exhibited severe pulmonary damage with a high wet-to-dry ratio, low partial pressure of oxygen, and low precursor surfactant protein C levels, which corresponded to the significant elevation of pro-inflammatory cytokines, β-hexosaminidase, and tryptase levels in serum and lung tissues. The severity of ALI progressed and maximized 8 h after OALT. Mast cell stabilization significantly inhibited the activation of mast cells, downregulated pro-inflammatory cytokine levels and translocation of NF-κB, and attenuated OALT-induced ALI.

**Conclusions:**

Mast cell activation amplified inflammation and played an important role in the process of post-OALT related ALI.

## Introduction

Liver transplantation is the most effective and efficient therapy for patients suffering from end-stage liver disease. However, the wide-spectrum post-operative complications of orthotopic liver transplantation (OLT) surgery, including technical, medical, and immunological complications, affect recipient outcome [1]. Acute lung injury (ALI) is one of the most severe post-operative complications that potentially contribute to mortality after liver transplantation [2]. We have previously reported that 58.2% of patients (91 patients in total) suffered from pulmonary complications after OLT, and about 27.5% of them suffered from ALI, and 5.5% of them endured adult respiratory distress syndrome (ARDS) [3]. Given that complicated risk factors are associated with ALI after OLT, effective and preventive strategies are inadequate [4], [5].

We and other researchers have revealed significant inflammation after intestine ischemia/reperfusion (I/R). The magnitude of inflammation was proportional to the severity of lung injury [6]–[10]. We have recently shown that rats suffering from ALI exhibited significant inflammatory response after OLT [11]. However, the mechanism underlying the activation process of remote organ inflammation after the original organ I/R is not clear.

Mast cells (MCs) originate from CD34^+^ pluripotent stem cells in the bone marrow and reside in different tissues for differentiation [12]. It has been reported that MCs may display potential role in asthma, multiple sclerosis, and I/R injury [13]. Tryptase in MCs participated in the process of inflammation [14]. Tumor necrosis factor α (TNF-α), which was triggered by MCs, played an important role in neutrophil recruitment to the inflammatory region and amplified local inflammation [15]. Until now, the functions of MC are not completely understood. We had shown that MCs were involved in ALI after small intestinal I/R [6], [8]. It is possible that MC activation participates in the process of activating remote organ inflammation.

Therefore, we hypothesize that severe pathophysiological variation during liver transplantation may trigger the degranulation of MCs, which amplifies inflammation and induces remote organ injury, especially ALI.

## Materials and Methods

### Animal Surgical Model and Experimental Design

This study was approved by the Institutional Animal Care and Use Committee of Sun Yat-sen University in Guangzhou, P.R. China, and followed the national guidelines for treatment of animals. Specific pathogen-free male Sprague–Dawley rats (280–320 g) were maintained spontaneous ventilation without intubation by inhaling 1%–3% isoflurane and 40% oxygen during surgery. The rats were then returned to room air and had free access to water after surgery. In order to determine the effect of MC stabilization on ALI, rats were pretreated with cromolyn sodium (25 mg/kg, i.v.) and ketotifen (1 mg/kg, i.v.) 15 min before OALT. The same volume of saline was used as a control. The doses and timing of cromolyn sodium and ketotifen which were used to pretreat the rats were described in previous publications [6], [16]–[18]. A sham group consisting of 16 rats was divided into four subgroups (with four rats in each subgroup) representing the post-sham surgical episode: sham 4, 8, 16, and 24 h. Meanwhile, an OALT group consisting of 32 rats was divided into four subgroups (with eight rats in each subgroup) representing the post-OALT period: post-OALT 4, 8, 16, and 24 h. We used 24 rats to investigate the effect of MC stabilization: eight rats were pretreated with cromolyn sodium, eight rats were pretreated with ketotifen, and eight rats were pretreated with the same volume of saline only.

The animal surgical model was described by Zhao and Zhou [19]. A middle incision was made on the abdomen. The left diaphragmatic vein, hepatoesophageal ligament vein, spleen vein, and right adrenal gland vein were ligated. Rats were given 50 U heparin (i.v.) 3 min before temporarily blockade of the portal vein, the hepatic artery, and infrahepatic venae cavae (IVC) with vascular clamps. A cannula was inserted 3 mm above the vascular clamp in the portal vein, followed by infusion with 2–3 mL of heparin saline (25 U/mL, 4°C) that impulsed the blood into systematic circulation. The suprahepatic venae cavae (SVC) was then temporarily blocked. The irrigation fluid outflow channel (1 mm) was cut on the IVC. Subsequently, the liver was irrigated with Plasma Lyte A (4°C, combined with 12.5 U/mL heparin at 100 mL/h velocity). The anhepatic phase remained for 20 min *in vivo*. The entrance and exit of the fluid were sutured after irrigation. Then, the liver was perfused by relieving the blockade of the portal vein, IVC, SVC, and hepatic artery. The liver and body were warmed by saline (38°C, 20 mL) which was infused into the abdominal cavity. About 1 mg of protamine sulfate (i.v.) was given before sewing the abdominal incision. For sham surgery, the rats were subjected to abdominal incision, portal vein dissociation, and suturing of the abdominal incision under anesthesia without the blockade of blood flow.

The accurate time of rebuilding liver reperfusion after the anhepatic phase was considered as the initiation of post-OALT, whereas the precise time of sewing up the abdominal incision was regarded as the initiation of post-operative episode in sham-group rats, since they did not experience an anhepatic phase. Subsequently, blood and lung tissues were collected to assess lung injury 4, 8, 16, and 24 h after surgery. The rats which were pretreated with MC stabilizers were executed 8 h after OALT. The experimental group design and animals used in each group were detailed in Table 1.

**Table 1:** Basic data of Sprague–Dawley rats and operative variables.

	Animals used in each group	Weight (g)	Duration of anhepatic phase (min)
	N	(mean ± SE)	(mean ±SE)
sham 4 h	4	295.00±7.91	0
sham 8 h	4	296.25±4.27	0
sham 16 h	4	297.50±6.61	0
sham 24 h	4	301.25±7.47	0
post-OALT 4 h	8	301.25±4.20	19.88±0.23
post-OALT 8 h	8	297.50±4.91	20.00±0.19
post-OALT 16 h	8	300.00±4.01	20.13±0.23
post-OALT 24 h	8	298.13±4.72	20.13±0.23
saline post-OALT 8 h	8	296.25±5.15	19.88±0.23
Cromonlyn post-OALT 8 h	8	295.63±3.95	20.00±0.19
Ketotifen post-OALT 8 h	8	293.75±4.41	20.00±0.27

Note: Body weight and anhepatic time duration in different groups show no statistical significance.

### Histopathology Analysis

The right upper lobes of lungs were immersed in 10% paraformaldehyde, dehydrated, and embedded in paraffin. The slices (4 µm thick) were deparaffinized and stained with hematoxylin and eosin (H&E) and sent to the pathologist who was blinded to the design details. The morphological changes of the lung were scored according to the criteria described by Hofbauer [20].

### Analysis of Arterial Blood Partial Pressure of Oxygen (PaO_2_)

Arterial blood samples were collected and immediately analyzed for PaO_2_ with a blood gas analyzer (i-STAT, Abbott Point, Ottawa, Ontario, Canada).

### Lung Wet-to-dry (W/D) Weight Ratio

The middle right lung lobe which was removed from the rat was measured for wet weight first. The dry weight was then recorded after the lung was placed in an oven at 80°C for 24 h. The lung W/D weight ratio was calculated by dividing the wet weight by the dry weight.

### Serum β-hexosaminidase Release Assay

Serum was collected to assess MC degranulation. 50 µl of serum was mixed with 50 µl of 4 mM p-nitrophenyl-N-acetyl-β-d-glucosaminide (dissolved with 0.2 M citric acid buffer, pH 4.5) (Merck, Germany), and the mixture was maintained at 37°C for 1 h. The reaction was terminated by adding 150 µl of 0.2 M glycine buffer (pH 10.7). Optical density (OD) was measured at 405 nm using a microplate reader (BioTel EXL-800, Winooski, Vermont, USA). The β-hexosaminidase release level was evaluated by (OD_serum_ – OD_test-blank_)/(OD_test-blank_ – OD_blank_)×100%.

### Immunohistochemistry (IHC) Analysis

After fixing in 10% paraformaldehyde, dehydrated, and embedded in paraffin, lung tissue slices (4 µm thick) were deparaffinized. The resident MCs were detected with anti-c-Kit (1∶100, Sigma, USA) antibody. Staining was done with avidin-coupled peroxidase, and counterstaining was done with hematoxyline.

### ELISA Kit for TNF-α, Interleukin (IL)-1β, and IL-6

TNF-α, IL-1β, and IL-6 concentrations in lung lysates and serum were quantified with commercial ELISA kits (KeyGen BioTech, China). All samples were measured in duplicate.

### Western Blot Analysis

Total proteins in lung tissue samples were ground and homogenized with a protein lysis solution (KeyGen BioTech, China). Nuclear and cytoplasmic proteins were separated according to the protocol described in Nuclear and Cytoplasmic Extraction Kit (Thermo Scientific, USA). Protein concentrations were measured with BCA method (KeyGen BioTech, China). The primary antibodies anti-pro-surfactant protein C (pro-SPC) (1∶2000, Merck Milipore, Germany), anti-MC tryptase (1∶500, Santa Cruz, USA), anti- nuclear factor-kappa B (NF-κB) p65 (1∶500, Santa Cruz, USA), anti-IkappaB-α (1∶1000, Cell Signaling Technology, USA), anti- Histone H2A (1∶500, Santa Cruz, USA) and anti-β-actin (1∶1500, Merck Milipore, Germany) were used to detect proteins levels. The optical density values were normalized to that of β-actin.

### Statistical Analysis

All values were expressed as the mean ± standard error (SE). Statistical comparisons were performed using SPSS 13.0 Statistical Software for Windows (SPSS, Chicago, IL, USA). Analyses included an ordinary *t*-test and repeated-measures ANOVA followed by the Bonferroni post-hoc test. Non-normal distributed data were compared using the Kruskal–Wallis test. When significant differences were found, the Mann–Whitney *U*-test was used to compare the two samples. Probability values less than 0.05 were considered statistically significant.

## Results

### Basic Data of OALT-treated Rats and Operative Variables

Table 1 shows no statistical significance in body weight (*p* = 0.197) and anhepatic phase duration time (*p* = 0.967).

### Orthotopic Autologous Liver Transplantation induced acute Lung Injury

**Figure 1:**
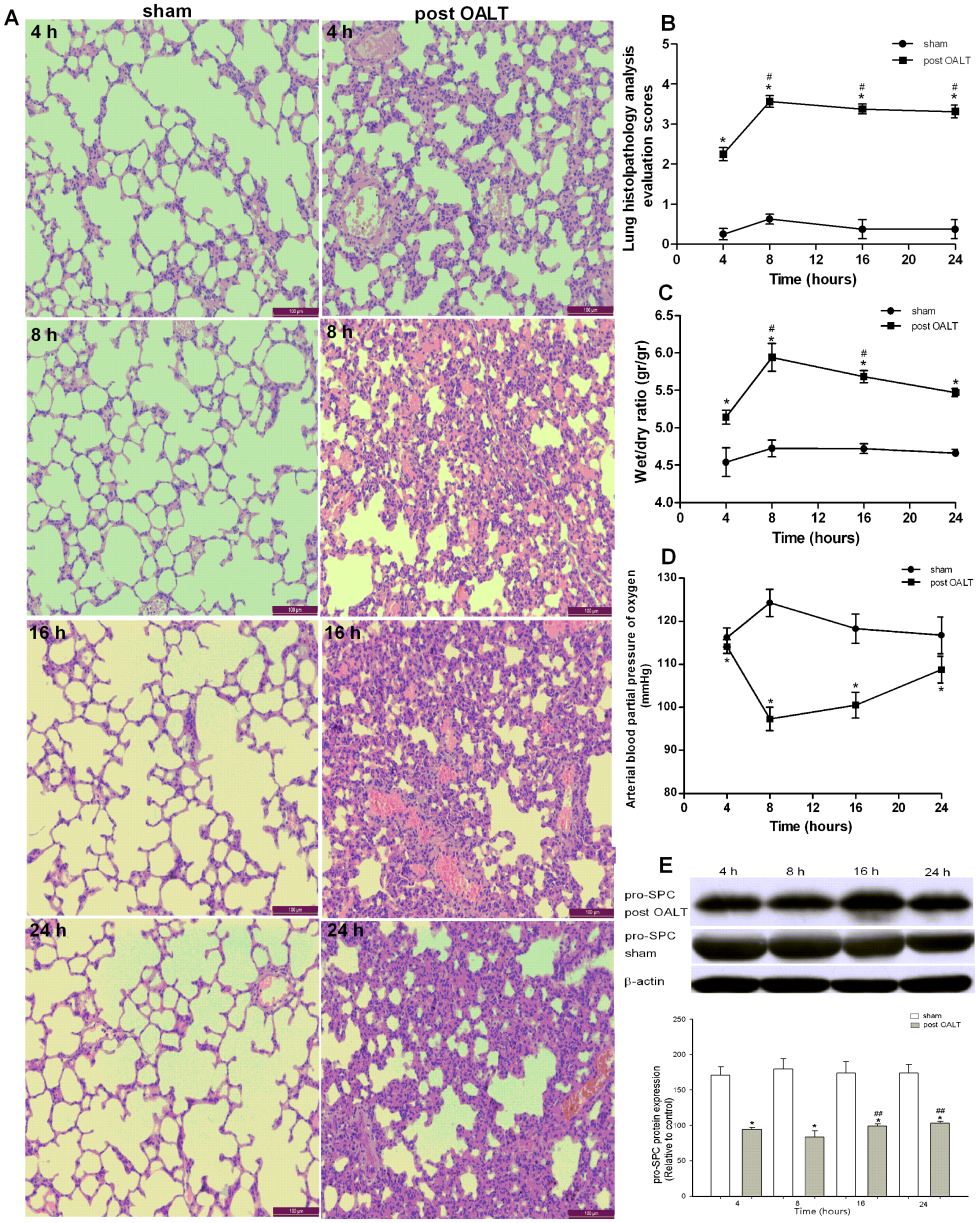
Orthotopic autologous liver transplantation (OALT) induced acute lung injury (ALI). (A) Lung histology morphology. Lung sections were stained with H&E and visualized at 200× magnification. (B) Lung histopathology analysis evaluation scores. (C) W/D weight ratio of the right middle lobe lung. (D) The arterial blood partial pressure of oxygen (PaO_2_). (E) Pulmonary precursor protein surfactant C (pro-SPC) expression and semi-quantitation of pro-SPC protein density for Western blot results by using Image software. Values are expressed as the mean ± SE. *p<0.01 vs. sham group, ^#^ *p*<0.05 vs. post-OALT 4 h. ^##^ *p*<0.05 vs. post-OALT 8 h.

Lung histopathological analysis revealed that the rats in the sham group exhibited normal structure and slight inflammatory cell infiltration. Meanwhile, the rats that received OALT displayed collapse of pulmonary architecture, severe infiltration of polymorphonuclear and mononuclear cells into intra-alveolar and interstitial spaces, and edematous interstitial space (Figure 1A). We found that the rats displayed severe pulmonary edema and pulmonary blood–gas barrier dysfunction with significantly elevated W/D (*p*<0.01) and decrease in PaO_2_ (*p*<0.01)_,_ accompanied with a significantly low pro-SPC (*p*<0.01) protein level in rats in the post-OALT period compared with the sham group. The results above reflected that lung injury maximized 8 h after OALT, which corresponded to the histopathological evaluation score (*p*<0.01) (Figures 1B–1E).

### Pro-inflammatory Cytokines IL-1β, IL-6, and TNF-α Levels

**Figure 2:**
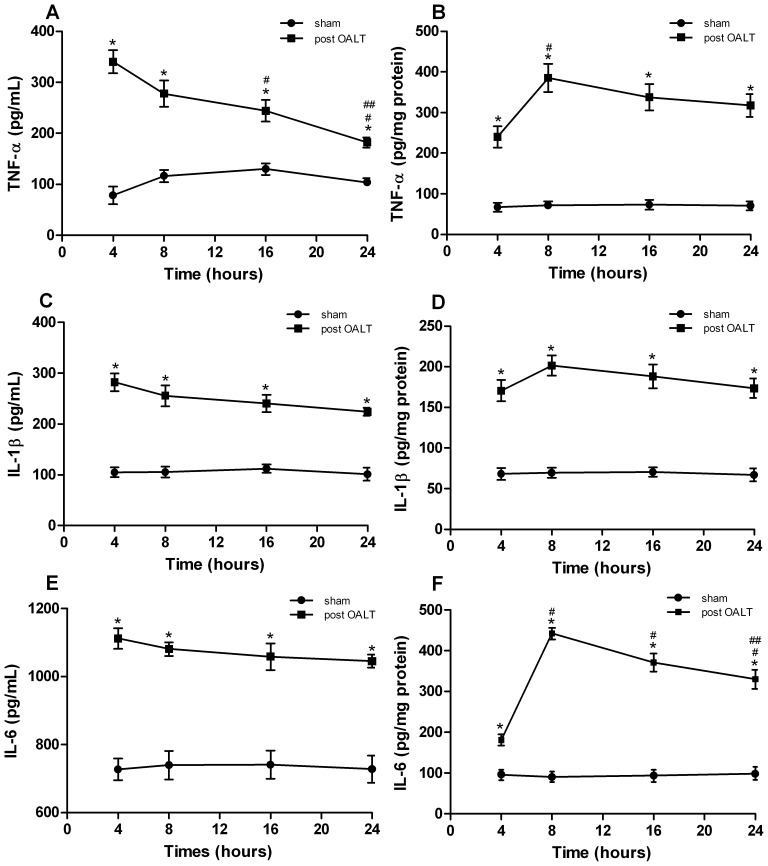
Upregulation of pro-inflammatory cytokines after OALT. Serum and lung tissue samples were accurately collected 4, 8, 16, and 24-α, IL-1β, and IL-6 levels by ELISA: (A) Serum TNF-α level. (B) Lung tissue TNF-α level. (C) Serum IL-1β level. (D) Lung tissue IL-1β level. (E) Serum IL-6 level. (F) Lung tissue IL-6 level. Values are expressed as the mean ± SE. *p<0.01 vs. sham group, ^#^ *p*<0.05 vs. post-OALT 4 h, ^##^ *p*<0.05 vs. post-OALT 8 h.

Compared with the sham group, the IL-6, IL-1β, and TNF-α concentrations in lung and serum significantly (*p*<0.01) increased in rats during post-OALT period (Figures 2A–2F). The IL-6, IL-1β, and TNF-α concentrations in serum reached the peak values 4 h after OALT, whereas these pro-inflammatory cytokines in lung tissue exhibited a burst 8 h after OALT. Compared with the cytokine level 4 h after OALT, TNF-α in serum significantly decreased 16 h (*p* = 0.016) and 24 h (*p*<0.01) after OALT, whereas the IL-6 and IL-1β concentrations in serum maintained a constantly high level. Compared with the pro-inflammatory cytokine levels in lung tissue 8 h after OALT, IL-6 sharply decreased 24 h after OALT (*p* = 0.002), while TNF-α and IL-1β in lung tissue did not significantly diminish 16 and 24 h after OALT.

### Activation of MC after OALT

**Figure 3:**
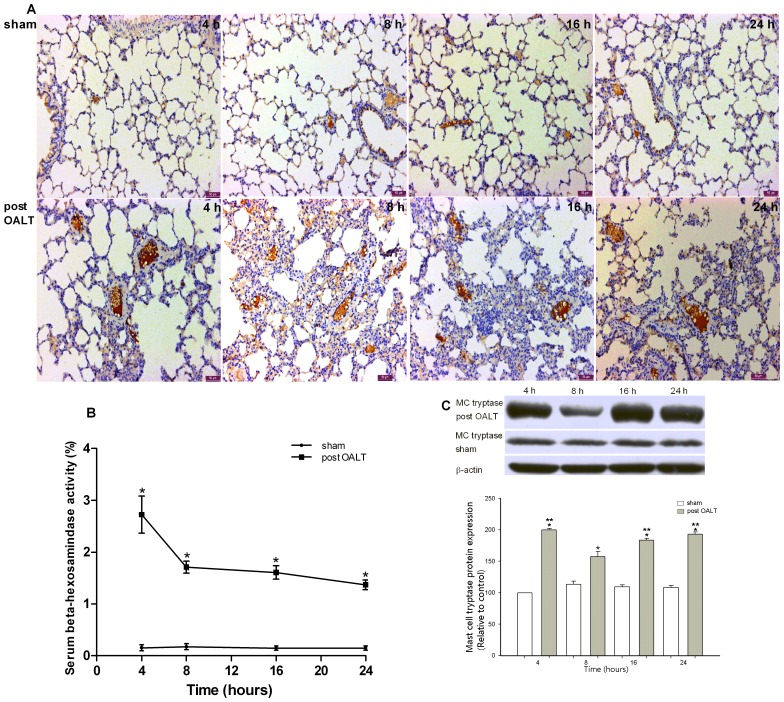
MCs activation analysis after OALT. MC immunohistochemical (IHC) analysis enabled the visualization of resident MCs in rats lung tissues by using CD117 antibodies. Serum samples of each animal were accurately collected 4, 8, 16, and 24 h after OALT to determine β-hexosaminidase release level, which indicated MC activation in rats after OALT. Tryptase in lung tissue was assessed by Western blot: (A) IHC for CD117-positive lung MCs after OALT. (B) β-hexosaminidase released in serum after OALT. (C) MC tryptase expression level after OALT. Values are expressed as the mean ± SE. *p<0.05 vs. sham group, **p<0.01 vs. post-OALT 8 h.

Analysis of β-hexosaminidase level was adopted to determine the activation of MC after OALT. Compared with the sham group, the serum β-hexosaminidase level significantly elevated after OALT (*p*<0.01), reached the maximum value 4 h after OALT (Figure 3B), and then gradually decreased in a time-dependent manner in the post-surgery period.

MC IHC and tryptase protein analysis enabled the visualization of resident MCs and semi-quantitation of the pro-inflammatory protease in lung. We successfully confirmed the resident MCs by using CD117 antibodies [21], [22]. Small amounts of MC population and degranulation, as well as low MC tryptase expression, were observed in the lungs of the sham group. The rats showed significant increase in MC population and degranulation with numerous vacuoles, as well as a significant increase in MC tryptase (*p*<0.01) in lung tissue 4 h after OALT (Figure 3A). MC tryptase significantly decreased at 8 h compared with the protease level 4 h after OALT (*p*<0.01). However, MCs initiated the newly synthesized MC tryptase 16 h after OALT and exhibited increased MC tryptase expression in lung (Figure 3C).

### MC Stabilization Alleviated ALI after OALT in Rats

We found that the rats experienced severe lung injury 8 h after OALT. Therefore, we chose 8 h after OALT as the crucial time point to investigate the therapeutic effect of MC stabilizers on ALI.

**Figure 4:**
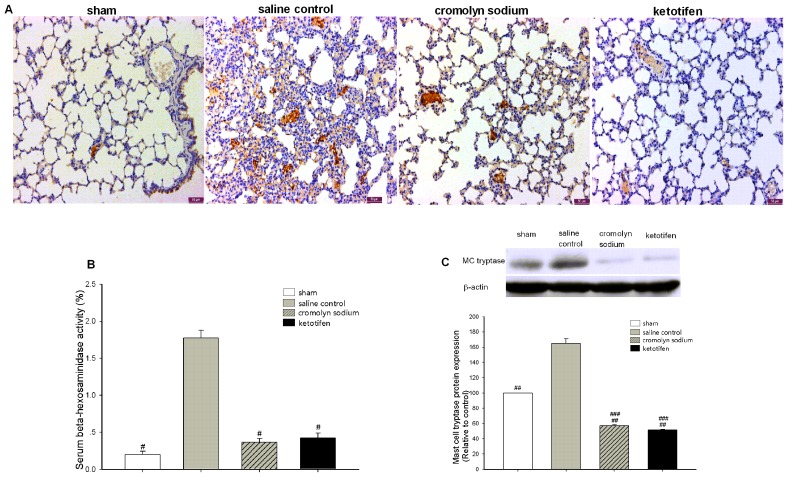
Downregulation of MC activation by MC stabilizers in rats 8 h after OALT. Lung tissues were collected 8β-hexosaminidase released. (A) IHC for CD117-positive lung MCs. (B) MC stabilization inhibited β-hexosaminidase release level in serum. (C) MC stabilization downregulated MC tryptase expression level 8 h after OALT. Values are expressed as the mean ± SE. ^#^ *p*<0.05 vs. saline control group, ^##^ *p*<0.01 vs. saline control group, ^###^ *p*<0.01 vs. sham group.

Compared with rats treated with saline only, both cromolyn sodium and ketotifen significantly inhibited the release of β-hexosaminidase into serum from MCs (*p*<0.01) (Figure 4B) and the degranulation of resident MCs and MC tryptase in lung tissue 8 h after OALT (*p*<0.01) (Figures 4A and 4C).

**Figure 5:**
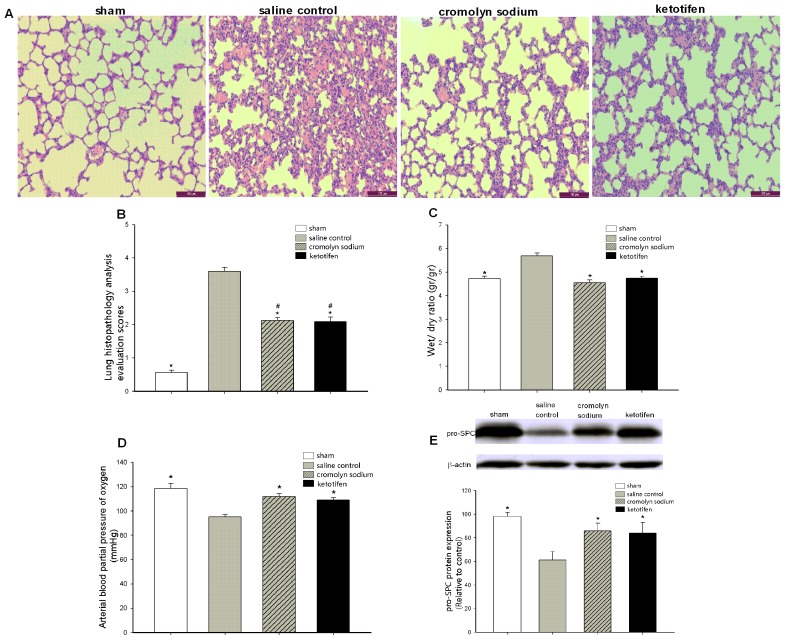
MC stabilizers alleviated ALI 8 (A) Lung histology morphology analysis after pretreatment of MC stabilizers. Lung sections were stained with H&E and visualized at 200× magnification. (B) Lung histopathology evaluation scores. (C) MC stabilization significantly decreased the W/D weight ratio of the right middle lobe lung 8 h after OALT. (D) MC stabilization improved the PaO_2_ of the rats 8 h after OALT. (E) MC stabilization increased pro-SPC expression level in rats 8 h after OALT. Values are expressed as the mean ± SE. *p<0.05 vs. saline control group, ^#^ *p*<0.05 vs. sham group.

**Figure 6:**
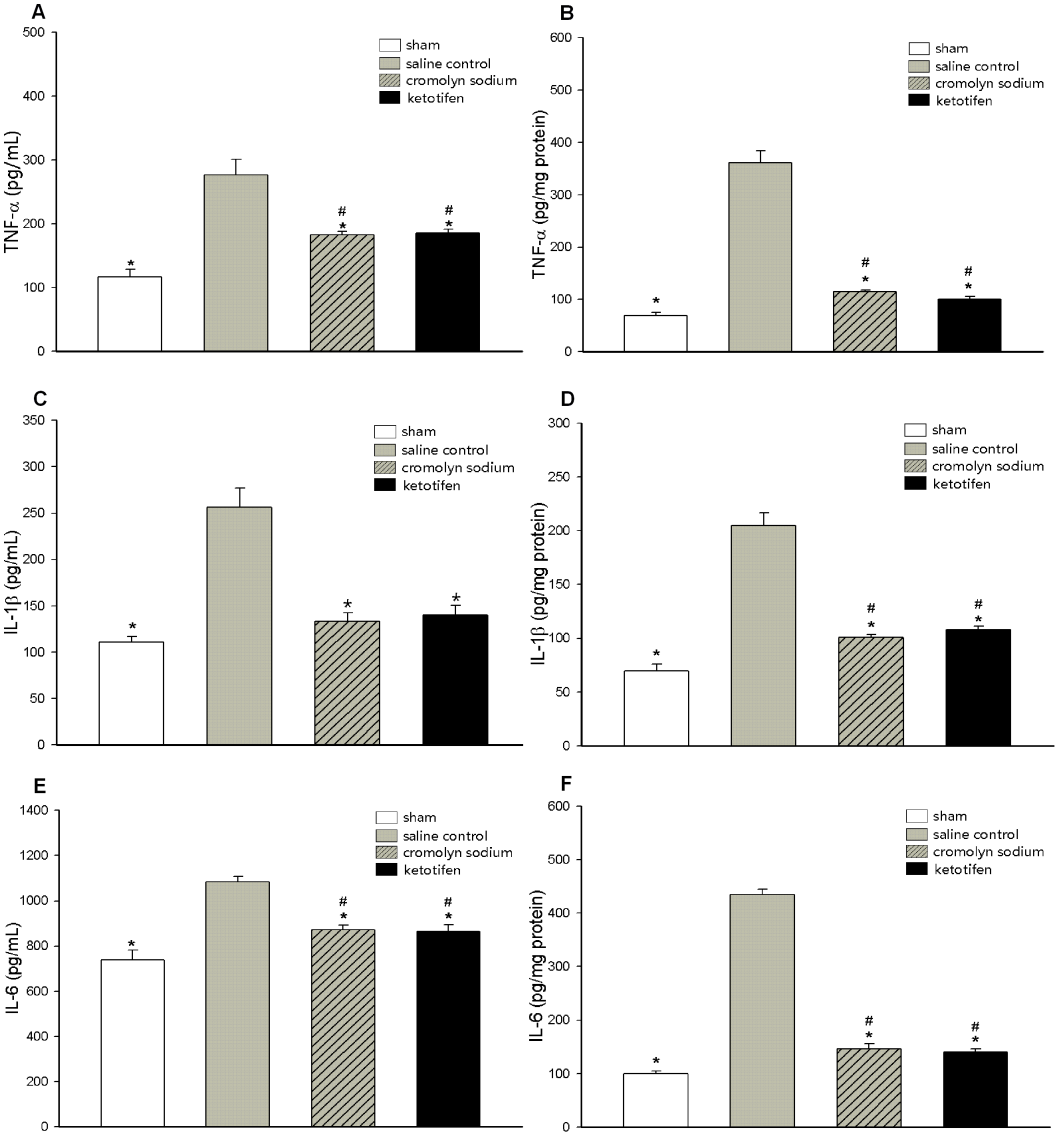
MC stabilization downregulated pro-inflammatory cytokines 8 h after OALT. (A) Serum TNF-α level. (B) Lung tissue TNF-α level. (C) Serum IL-1β level. (D) Lung tissue IL-1β level. (E) Serum IL-6 level. (F) Lung tissue IL-6 level. Values are expressed as the mean ± SE. *p<0.05 vs. saline control group, ^#^ *p*<0.05 vs. sham group.

**Figure 7:**
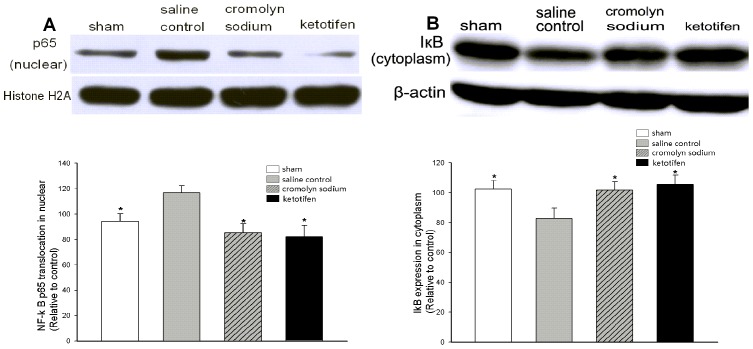
MC stabilizers inhibited the translocation of NF- κ**B p65 to the nucleus.** Lung tissue transcription factor NF-κB p65 expression in nucleus and IκB expression in cytoplasm were assessed by Western blot 8 h after OALT. (A) NF-κB p65 expression in nucleus. (B) IκB expression in cytoplasm. Values are expressed as the mean ± SE. *p<0.05 vs. saline control group.

Lung tissues of each animal were accurately assembled 8 h after OALT to evaluate the effect of MC stabilization on ALI evoked by transplantation. Both cromolyn sodium and ketotifen pretreatments alleviated ALI after OALT (Figure 5). Compared with the saline control group, fewer inflammatory cell infiltration and less edematous interstitial space were observed in lung tissue, which showed normal pulmonary architecture in rats pretreated with MC stabilizers. Histological morphology evaluation analysis revealed that both cromolyn sodium (*p* = 0.006) and ketotifen (*p* = 0.006) greatly alleviated ALI after OALT, accompanied with a significant decrease in W/D (*p*<0.01) and a significant increase in PaO_2_ (*p*<0.01) and pro-SPC expression (*p*<0.05) (Figures 5B–5E). The IL-1β, IL-6, and TNF-α levels in circulation and local region were profoundly downregulated 8 h after OALT in rats pretreated with MC stabilizers (*p*<0.05) (Figure 6). Meanwhile, we revealed that MC stabilization significantly decreased the degradation of IκB-α (*p* = 0.032, cromolyn sodium vs. saline control; *p* = 0.011, ketotifen vs. saline control) in cytoplasm and the translocation of NF-κB p65 (*p* = 0.003, cromolyn sodium vs. saline control; *p* = 0.002, ketotifen vs. saline control) to nucleus compared with rats pretreated with saline only (Figure 7).

## Discussion

Pulmonary injury after OLT increased post-operative mortality [23]. The unrestrained inflammation contributed to ALI development [24]. Orthotopic autologous liver transplantation is not exactly the same as liver transplantation that requires original liver depletion and new liver transplantation. The OALT model is superior in emphasizing pathophysiological variation during liver I/R as well as in exploring the role of MC in liver transplantation and I/R-mediated ALI without interference of immuno-activities between grafts and hosts. Moreover, we did not use a respirator for mechanical ventilation during surgery because ventilation-related ALI may be involved in the research process. We demonstrated that rats endured ALI after OALT, as manifested by severe pulmonary edema and inflammatory cell infiltration in lungs, increased W/D, and decreased pro-SPC and PaO_2._ The rats suffered from the severest lung injury 8 h after OALT, though ALI was gradually relieved at 16 and 24 h, the intrinsic repair cannot restore this injury. MC stabilization greatly inhibited the systemic and local inflammation and alleviated OALT-induced ALI.

Liver I/R injury initiated the explosion of pro-inflammatory cytokines, which may trigger remote organ injury. It had been reported that the IL-1 family members increased significantly after liver injury. IL-1β elevated in serum and then in lung tissue, and IL-1β can also activate the NF-κB pathway [25]. Downregulation of the IL-1β level by RNA interference greatly reduced NF-κB activation within livers and lungs, which greatly relieved ALI induced by liver I/R [26], [27]. TNF-α was not a direct chemoattractant after liver I/R, but it induced chronic inflammation through the NF-κB pathway [28]. TNF-α has been indentified as an important pro-inflammatory cytokine which could induce the degradation of IκBα and translocation of NF-κB to the nucleus [29]. High IL-6 levels were observed in patients and animal models suffering from ALI or ARDS [30], [31]. Translocation of NF-κB, which was stimulated by TNF-α and IL-1β, enhanced IL-6 production [32]. In our study, IL-1β, TNF-α, and IL-6 significantly increased in circulation and lungs in rats with ALI after OALT. The magnitude of lung injury was proportional to the IL-1β, TNF-α, and IL-6 levels in lung tissue. These results suggested that pro-inflammatory cytokines flooding after liver I/R played critical roles in activating the NF-κB pathway and amplifying lung inflammation and injury. The pretreatment of MC stabilizers (either cromolyn sodium or ketotifen) significantly inhibited MC activation and pro-inflammatory cytokine levels in serum and lung, and profoundly decreased NF-κB p65 subunit translocation to the nucleus in lung 8 h after OALT. This finding indicated that MC activation may be involved in the mechanism of pro-inflammatory cytokine release and remote organ injury. However, how MCs modulate pro-inflammatory cytokine expression after OALT requires further investigations.

Cromolyn sodium and ketotifen are considered as effective agents for treating allergic reactions for decades. The role for alleviation the allergen-induced response and late-phase responses in airways is well established [33], [34]. However, the precise mechanism of cromolyn sodium is not fully elucidated. The cromolyn sodium-binding protein expressed in MCs may inhibit calcium flux into the cell before antigen and non-antigen challenge [35], [36]. Ketotifen, which inhibits histamine receptors, can effectively prevent mediators to be released from MCs and treat allergic diseases in experimental and clinical trials [17], [37]–[43].

MCs obtain their phenotypes after residing in different environments. Thus, the heterogeneity of MCs confers difficulty in illustrating precise functions [13]. Pre-synthesized substances such as histamine, chymase, tryptase, carboxipeptidase A, and TNF-α were stored in granules and immediately released after MC activation, and the various mediators exerted the potential capacity of activating inflammation [13]. Apart from IgE, different stimuli can trigger the activation of MCs, and MCs released tremendous mediators within a few minutes [44]–[47]. TNF-α, IL-6, and IL-1β were among the cytokines which were *de novo* synthesized after MC activation [48]. Moreover, IL-6, which was induced by TNF-α, IL-1β, and platelet-activating factor (PAF), provided positive feedback on MC activation and migration [49]. TNF-α, triggered by the degranulation of MCs, induced ICAM-1 overexpression and facilitated the migration of neutrophils through the endogenously generated or exogenous chemokines KC/CXCL1 and LIX/CXCL5 [15]. MC tryptase was considered as one of the ligand enzymes for protease-activated receptor 2 (PAR-2) which was abundantly expressed in the airways [50]. Tryptase-mediated PAR-2 activation induced various lung disorders [51]. We have shown that tryptases may provocate lung inflammation and ALI by activating PAR-2 [6]. The inflammatory role of PAR-2 was clarified in most publications. PAR-2 activation greatly enhanced the eotaxin level, which resulted in eosinophil infiltration and airway inflammation. By contrast, in PAR-2-deficient mice (PAR2^−/−^), accumulation of eosinophils and eotaxin content were significantly downregulated [52]. By using PAR-activating peptides, significant lung inflammation, extravascular lung water, and high airway resistance were observed in wide-type mice, whereas deprivation of sensory neurons by capsaicin significantly diminished PAR2-mediated pulmonary inflammation and complications [53]. In addition, the MC-derived lipid mediators arachidonic acid metabolites (e.g., prostaglandin _2_ (PGD_2_), leukotriene A_4_ (LTA_4_), leukotriene B_4_ (LTB_4_), and leukotriene C_4_ (LTC_4_)) and PAF that were synthesized following the activation of phospholipase (PL)A_2_ after MC activation [54] might promote pulmonary inflammation. LTB_4_ had been identified as an important chemoatractant for leukocytes, especially esoinophils, which greatly contributed to airway inflammation and asthma [55]. PAF possessed a wide spectrum of biological activities [56], while lung inflammation was greatly involved in the PAF pathway. Several experimental models showed that PAF could activate lung inflammation, which was evidenced by increasing vascular permeability and pulmonary edema [57], [58]. Anti-PAF treatment inhibited lung inflammation after liver transplantation by reducing adhesion molecules and the interaction with leukocytes and endothelium [59]. PGD_2_ potentiated pulmonary eosinophilic inflammation by the activation of chemoattractant receptor homologous molecules expressed in Th2 cells (CRTH2) and accumulation of eosinophil from circulation to the airway, whereas CRTH2 antagonist effectively controlled the airway inflammation [60]. In the present study, we confirmed the MC activation in rats experiencing OALT, with significant release of β-hexosaminidase in serum and MC tryptase expression in lung tissues. MC stabilization significantly inhibited MC activation and the mediators related lung injury after OALT.

In summary, the results substantiated our hypothesis that MC activation which was triggered by greatly fluctuating hemodynamic change and severe liver I/R injury contributed to OALT-induced ALI. Flooded mediators which were released from MCs may directly generate epithelial injury and indirectly cause accumulation of inflammatory cells and translocation of NF-κB to the nucleus in lung, which profoundly amplified lung inflammation after OALT. MC stabilization effectively and efficiently downregulated MC activation and alleviated OALT-induced ALI.
